# Supplementary material for: HPLC–DAD Analysis, SFE-CO2 Extraction, and Antibacterial Activity on Bioactive Compounds from Mosla chinensis Maxim
Source: Molecules. 2023 Nov 23;28(23):7724. doi: 10.3390/molecules28237724 (PMC10707893; doi:10.3390/molecules28237724)
Supplement: Supplementary file 1 [file molecules-28-07724-s001.zip › molecules-2722495-supplementary.pdf]

Supplementary material

HPLC-DAD analysis, SFE-CO_2 extraction and antibacterial activity on bioactive compounds from *Mosla chinensis* maxim

Rui-xi Gao [1,†], Bingchen Han [2,†] Yanfeng Zeng [1], Linchuang Shen [1], Xinqiao Liu [1], Qiang Wang [1], Maochuan Liao [1, 3,*,] and Jun Li [1,4,*]

[1] School of Pharmacy, South-Central Minzu University, Wuhan 430074, China

[2] College of Life Sciences, South-Central Minzu University, Wuhan 430074, China

[3] School of Life Sciences, Wuchang University of Technology, Wuhan 430223, China

[4] Ethnopharmacology Level 3 Laboratory, National Administration of Traditional Chinese Medicine, South-Central Minzu University, Wuhan 430074, China

† These authors contributed equally to this work.

* Correspondence: sippr1976@163.com (M. Liao), lijun-pharm@hotmail.com (J. Li).

Contents

Supplementary figure:

1	Thin-layer chromatography (TLC) results for *M. chinensis*.	PP. 3
2	SFE-CO_2 pilot experiment samples of *M. chinensis* (10 t).	PP. 4
3	Antibacterial zone results for *M. chinensis.*	PP. 5

Supplementary table:

1	Production of *M. chinensis* and related planting information of the cultivated species.	PP. 6
2	First-hand market price (USD) of *M. chinensis* (2014-2023).	PP. 7

Fig. S1.

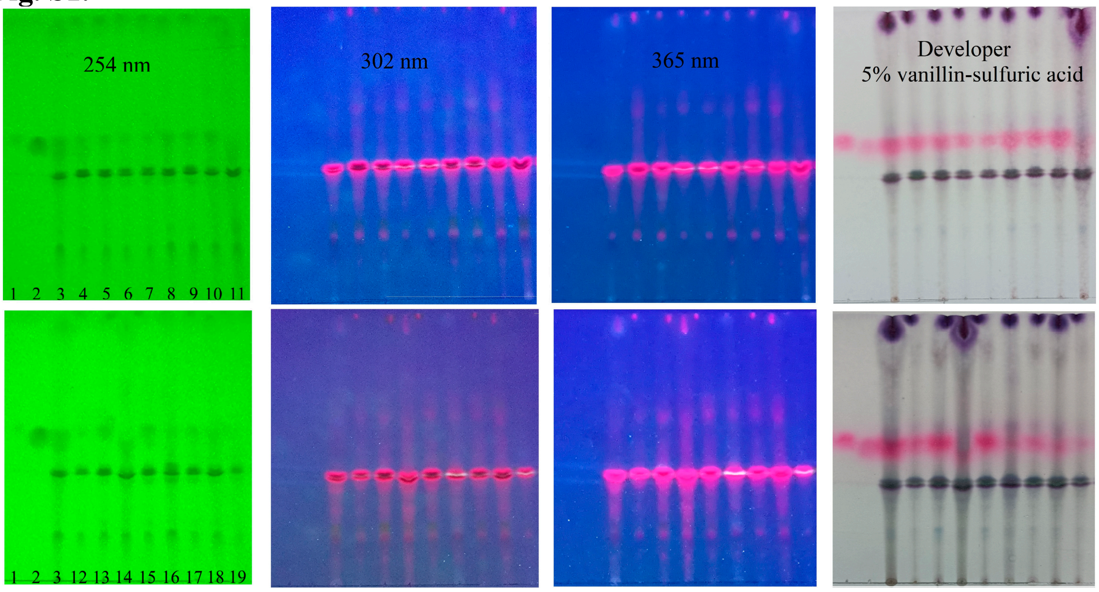

Thin layer chromatography (TLC) results for bioactive compounds from *M. chinensis*.

Two main compounds in *M. chinensis*, thymol (**1**), carvacrol (**2**), together with the standard plant (**3**, *M. chinensis*, identified by National Institutes for Food and Drug Control, Beijing, China) and plants from 16 regions (**4-19**) were analysed and distinguished by UV (254, 302, 365 nm) and chromogenic developer (5% vanillin-sulphuric acid). The mobile phase was hexane:toluene:acetone:acetic acid (2:8:0.5:0.5, v/v).

16 regions were (**4-19**): Anguo (Hebei, **4**); Nanjing (Jiangsu, **5**); Bozhou (Anhui, **6**); Huoshan (Anhui, **7**); Chengdu (Sichuan, **8**); Shennongjia (Hubei, **9**); Panan (Zhejiang, **10**); Quzhou-1 (Zhejiang, **11**); Quzhou-2 (Zhejiang, **12**); Yichun (Jiangxi, **13**); Sanming (Fujian, **14**); Kunming (Yunnan, **15**); Guangzhou (Guangdong, **16**); Jieyang (Guangdong, **17**); Meizhou (Guangdong, **18**) and Yulin (Guangxi, **19**).

Fig. S2.

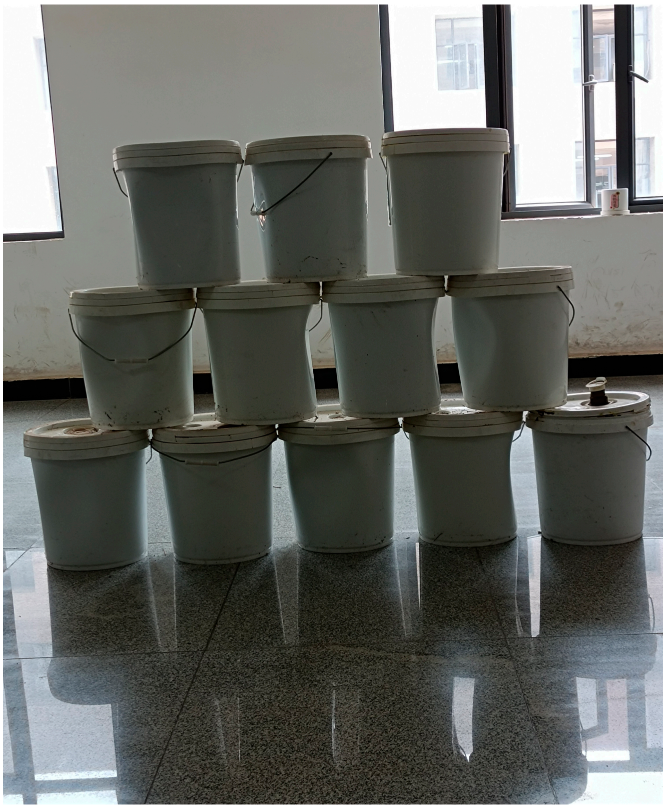

SFE-CO_2 pilot extraction of *M. chinensis*. 10 t plants were used for extraction, yielding 334 kg samples.

Fig. S3. Antibacterial zone results for *M. chinensis*, the concentration was 32 μg/mL.

Name	Antibacterial zone	Name	Antibacterial zone
Gardnerella vaginalis	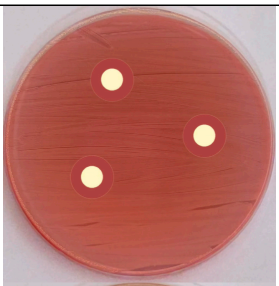	*Helicobacter pylori*	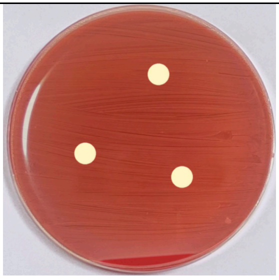
Propionibacterium acnes	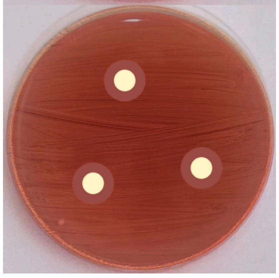	*Malassezia furfur*	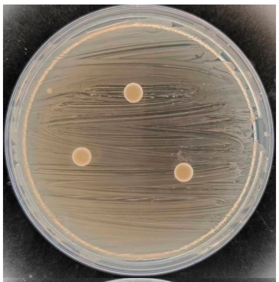
Staphylococcus aureus	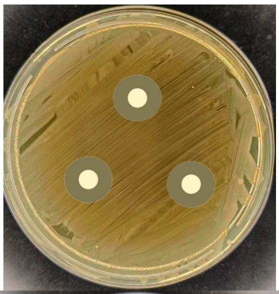	*Aspergillus flavus*	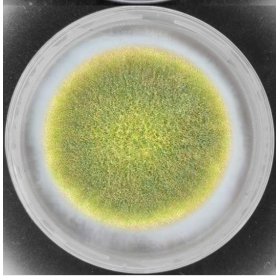
Staphylococcus epidermidis	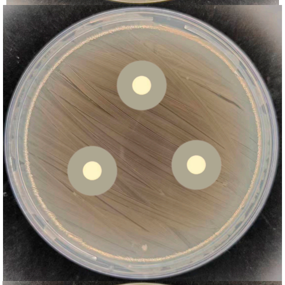	*Epidermophyton floccosum*	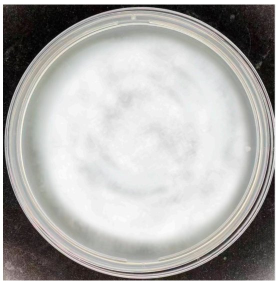
methicillin-resistant *Staphylococcus aureus*	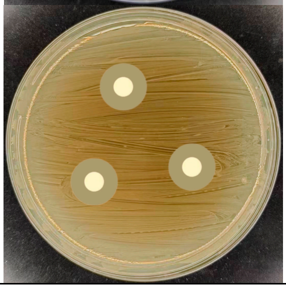		

Table S1. Estimated production of *M. chinensis* and the planting information for the cultivated species.

Estimated total plant production	1900 t (2021), 2200 t (2020)
Planting information on cultivated *M. chinensis* in Jiangxi Province and Shaoyang city, Hunan province, China (2022)	
Planting scale	2000 mu, 133.4 hectares, 1.33 km^2 (1 mu = 0.0667 hectares)
Production	300 kg/mu
Estimated total production	600 t

Table S2. First-hand market price (USD) of *M. chinensis*. Those data were collected from the herb markets in Anhui, Hubei, Hunan, Jiangxi and Hebei provinces, China.

Time (Year-Month)	Price ($)/kg
2014-12	0.9117
2015-01	0.9117
2015-05	0.9117
2015-09	0.8682
2015-10	0.7235
2016-06	0.7235
2016-09	0.7235
2017-01	0.7235
2017-06	0.7235
2017-12	0.7235
2018-01	0.6512
2018-05	0.6512
2018-10	0.6512
2018-11	0.7959
2019-04	0.7959
2019-12	0.7959
2020-01	0.7959
2020-06	0.7959
2020-12	0.7959
2021-06	0.7959
2021-12	0.7959
2022-04	0.7959
2022-09	0.7959
2022-10	1.0130
2022-11	1.0130
2023-01	1.0130
